# Red Chinese Cabbage Transcriptome Analysis Reveals Structural Genes and Multiple Transcription Factors Regulating Reddish Purple Color

**DOI:** 10.3390/ijms21082901

**Published:** 2020-04-21

**Authors:** Jana Jeevan Rameneni, Su Ryun Choi, Sushil Satish Chhapekar, Man-Sun Kim, Sonam Singh, So Young Yi, Sang Heon Oh, Hyuna Kim, Chang Yeol Lee, Man-Ho Oh, Jhongchul Lee, Oh Ha Kwon, Sang Un Park, Sun-Ju Kim, Yong Pyo Lim

**Affiliations:** 1 Molecular Genetics and Genomics Laboratory, Department of Horticulture, College of Agriculture and Life Science, Chungnam National University, Daejeon 34134, Korea; 2 Department of Biological Sciences, College of Biological Sciences and Biotechnology, Chungnam National University, Daejeon 34134, Korea; 3 Kwonnong Seed Co., 186 Pungnyeon-ro, Heungdeok-gu, Cheongju 28394, Korea; 4 Department of Crop Science, College of Agriculture and Life Science, Chungnam National University, Daejeon 34134, Korea; 5 Department of Bio-Environmental Chemistry, College of Agriculture and Life Science, Chungnam National University, Daejeon 34134, Korea

**Keywords:** anthocyanins, anthocyanin biosynthetic genes, *cis*-regulatory motifs, DEGs, network analysis, qRT-PCR, reddish purple Chinese cabbage, transcriptome, transcription factors

## Abstract

Reddish purple Chinese cabbage (RPCC) is a popular variety of *Brassica rapa* (AA = 20). It is rich in anthocyanins, which have many health benefits. We detected novel anthocyanins including cyanidin 3-(feruloyl) diglucoside-5-(malonoyl) glucoside and pelargonidin 3-(caffeoyl) diglucoside-5-(malonoyl) glucoside in RPCC. Analyses of transcriptome data revealed 32,395 genes including 3345 differentially expressed genes (DEGs) between 3-week-old RPCC and green Chinese cabbage (GCC). The DEGs included 218 transcription factor (TF) genes and some functionally uncharacterized genes. Sixty DEGs identified from the transcriptome data were analyzed in 3-, 6- and 9-week old seedlings by RT-qPCR, and 35 of them had higher transcript levels in RPCC than in GCC. We detected *cis-*regulatory motifs of MYB, bHLH, WRKY, bZIP and AP2/ERF TFs in anthocyanin biosynthetic gene promoters. A network analysis revealed that MYB75, MYB90, and MYBL2 strongly interact with anthocyanin biosynthetic genes. Our results show that the late biosynthesis genes *BrDFR, BrLDOX, BrUF3GT, BrUGT75c1-1, Br5MAT, BrAT-1,* *BrAT-2, BrTT19-1,* and *BrTT19-2* and the regulatory MYB genes *BrMYB90, BrMYB75,* and *BrMYBL2-1* are highly expressed in RPCC, indicative of their important roles in anthocyanin biosynthesis, modification, and accumulation. Finally, we propose a model anthocyanin biosynthesis pathway that includes the unique anthocyanin pigments and genes specific to RPCC.

## 1. Introduction

Introgression breeding is an important traditional breeding technique for transferring key agronomic traits between two distinct species [1]. Using this technique, improvements have been made to many *Brassica* traits, such as disease resistance, male sterility, seed color, oil quality traits, and other morphological traits [1,2]. Some purple Brassicaceae lines have also been generated [3,4]. Red Chinese cabbage (*Brassica rapa* ssp. *pekinensis* L.) is reddish purple in color and rich in anthocyanins. This vibrantly colored variety is a popular addition to salads and it has important antioxidant properties [4].

Anthocyanins are a class of secondary metabolites that are synthesized through the phenylpropanoid pathway [5]. These water-soluble compounds with red, purple, or blue colors are synthesized in the cytosol and stored in the vacuole [6]. Anthocyanins play crucial roles in reducing damage from, and in defense responses against, abiotic stresses such as ultraviolet exposure, wounding, high light, chilling, pollution, osmotic stress, and nutrient deficiency, as well as biotic stresses such as pathogen infection [7].

The important structural genes in the anthocyanin biosynthetic pathway are those encoding phenylalanine ammonia-lyase (PAL), cinnamate 4-hydroxylase (C4H), 4-coumaroyl CoA ligase (4CL), chalcone synthase (CHS), chalcone isomerase (CHI), flavanone 3-hydroxylase (F3H), flavonoid 3′-hydroxylase (F3′H), dihydroflavonol 4-reductase (DFR), leucoanthocyanidin dioxygenase (LDOX), UDP-flavonoid glucosyl transferase (UFGT) and glutathione S-transferase (GST) [8]. The late anthocyanin biosynthesis genes are regulated by a complex of transcription factors (TFs), MYB-bHLH-WD40, known as the MBW complex. In vivo assays in *Antirrhinum* revealed that a flower-specific MYB protein activates the transcription of genes involved in phenylpropanoid biosynthesis [9,10]. Four MYB TFs, encoded by *PAP1/MYB75, PAP2/MYB90, MYB113*, and *MYB114*, have been identified to control anthocyanin biosynthesis in vegetative tissues of *Arabidopsis* [11,12]. Previous biochemical and genetic studies have shown that TTG1 (WD40), GL3/EGL3/TT8 (bHLH) and PAP1/PAP2/MYB113/MYB114 (MYB) are components of potential WBM complexes that activate anthocyanin biosynthesis [13,14]. Interestingly, recent studies on proanthocyanidin and anthocyanin biosynthesis pathways in *Arabidopsis* and *Petunia* suggest that WRKY TFs regulate color accumulation along with the MBW complex [10,15]. Similarly, studies on bZIP-TFs revealed the mechanisms by which they regulate anthocyanin accumulation in apples [16,17].

Secondary metabolites are abundant in Chinese cabbage. Previous studies have shown that some secondary metabolites are specific to particular species, cultivars, or varieties. Such metabolites are usually detected by high performance liquid chromatography (HPLC) coupled with liquid chromatography-tandem mass spectrometry (LC-MS/MS) [4,18,19,20]. Recent metabolic profiling studies have demonstrated that cyanidin derivatives are highly accumulated in *Brassica* species [18,21,22,23]. It is difficult to identify all the genes related to specific traits in a plant system, but transcriptome sequencing allows for the prediction of functional genes in the plant genome. Several RNA-seq studies have been conducted for *Brassica* species, and their results have led to the identification of sets of genes that are expressed under various conditions and/or in certain genotypes. This information is very useful for the functional characterization of target genes [24,25,26,27].

Reddish purple Chinese cabbage (RPCC) is a variety of *B. rapa*. It is an economically important leafy vegetable that is widely cultivated and consumed in East Asian countries, especially Korea, due to its health promoting properties [28]. To date, the underlying molecular mechanisms and the genes regulating the anthocyanin pigments responsible for its vivid red color are unexplored, and may differ from those reported previously for other *Brassica* lines. In this study, we used a next generation sequencing (NGS) based RNA-sequencing approach to identify a novel set of genes involved in anthocyanin biosynthesis, and the regulation of this pathway, in a red Chinese cabbage variety. A comprehensive analysis of this plant including computational, leaf chemotype, and expressional abundance analyses shows the significance of this variety.

## 2. Results

### 2.1. Estimation of Anthocyanin Content in RPCC and GCC Leaf Samples

**Figure 1:**
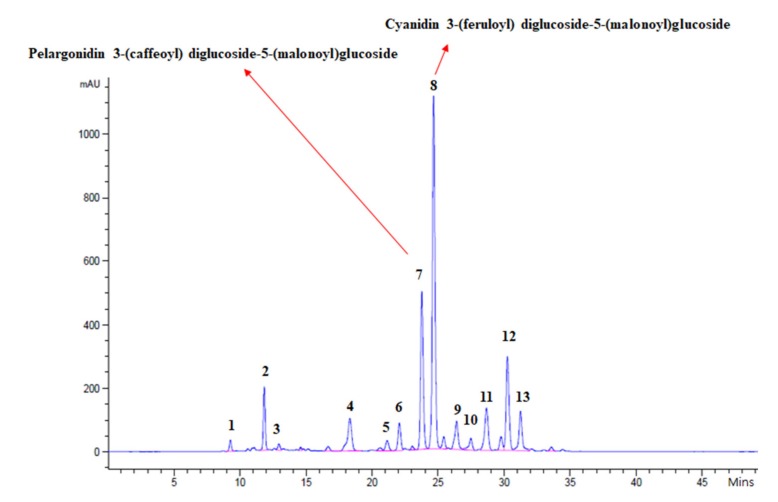
HPLC chromatogram of anthocyanin pigments detected in leaf extract of reddish purple Chinese cabbage at 520 nm. Horizontal axis shows retention time (min); vertical axis indicates strength of the peak (mAU).

The red color is a distinguishing feature of some *Brassica* species, and is due to the accumulation of anthocyanins [29]. To determine the types of anthocyanins that confer color in RPCC, we analyzed anthocyanins in the innermost and outermost leaves of 9-week-old GCC and RPCC plants (Table 1 and Table S1) by HPLC-MS/MS. We detected 13 anthocyanin pigments, 12 of which were derivatives of cyanidin. The other one was a pelargonidin derivative. The most abundant pigment in the innermost leaves of RPCC was cyanidin 3-(feruloyl) diglucoside-5-(malonoyl) glucoside (peak 8) with a concentration of 12.63 ± 1.37 mg/g dry weight, followed by pelargonidin 3-(caffeoyl) diglucoside-5-(malonoyl) glucoside (peak 7) with a concentration of 5.66 ± 0.60 mg/g dry weight (Figure 1; Table 1 and Table S1). In the outermost leaves of RPCC, the most abundant pigments were cyanidin 3-(feruloyl) diglucoside-5-(malonoyl) glucoside and cyanidin 3-*O*-(sinapoyl)(feruloyl) diglucoside-5-*O*-(malonyl) glucoside (Figure 1; Table 1 and Table S1). The total amount of anthocyanins was 32.31 ± 2.84 mg/g dry weight in the innermost leaf and 10.17 ± 1.69 mg/g dry weight in the outermost leaf of RPCC. No anthocyanins were detected in the GCC leaves (Table 1). Interestingly, the RPCC pigment peaks 8 and 7 and a few other pigments identified in this study (Table S1) had not been reported previously. This result indicates that cyanidin 3-(feruloyl) diglucoside-5-(malonoyl) glucoside and pelargonidin 3-(caffeoyl) diglucoside-5-(malonoyl) glucoside are specific to this variety of RPCC, and contribute to its color.

### 2.2. RNA-Sequencing of RPCC and GCC Samples

To identify differences in gene transcription between RPCC and GCC, we obtained and sequenced 53,127,646 (GCC) and 48,179,516 (RPCC) total reads, making up 5.37 Gb (giga bases) and 4.87 Gb, respectively (Table 2) with a GC content of 47.6% and 47.2%, respectively. After filtering, 38,611,432 and 34,924,846 clean reads were obtained for the GCC and RPCC samples, respectively, with Q30 values of 94.4% and 94.1%, respectively. A total 32,395 non-redundant transcripts were identified with varying lengths, most in the range of 501 to 1000 nucleotide bases, followed by 200–500 and 1001–1500 nucleotide bases (Figure S1a). Among the identified transcripts, 90.2% were annotated to the following public databases: NR-Viridiplantae (88.61%)¸ Phytozome (88.05%), UniProtKB–Viridiplantae (85.31%), KOG (84.06%), GO (83.72%), InterProscan (68.43%), and KEGG (18.87%) (Figure S1b).

### 2.3. Identification of DEGs

**Figure 2:**
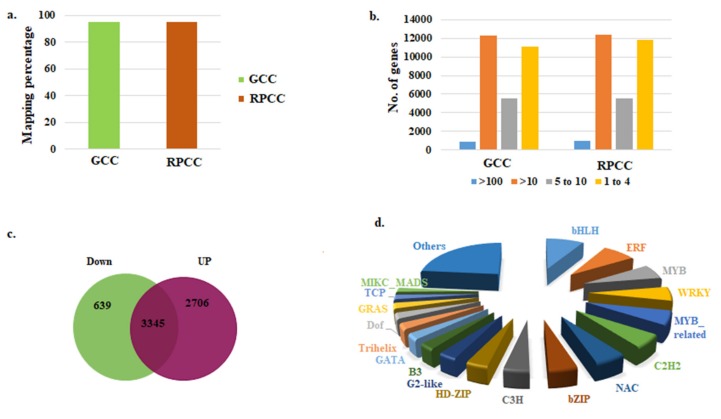
RNA-seq data for reddish purple Chinese cabbage. (**a**) Percentage of transcripts mapped to reference genome; (**b**) Gene expression values (RPKM); (**c**) Differentially expressed genes (DEGs) between two genotypes [green (GCC) and reddish purple (RPCC)]; (**d**) Transcription factor families identified in the transcriptome.

The cDNA libraries of RPCC and GCC were mapped to the *B. rapa* reference genome with coverage of 95.2% and 94.66%, respectively (Figure 2a). Among the mapped and annotated DEGs, the highest proportion had RPKM (reads per kilobase per million mapped reads) values of >10, followed by RPKM values of 1 to 4 (Figure 2b). Among the predicted 3345 DEGs, 2706 were up-regulated and 639 were down-regulated in RPCC vs. GCC (Figure 2c; Table S2 and Table S3). Of them, 643 of the up-regulated DEGs and 354 of the down-regulated DEGs between RPCC and GCC samples were not functionally characterized (Table S2 and Table S3). The DEGs included many ABGs. A Bland–Altman (MA) plot was constructed to show the differentiation of gene expression between the two samples by plotting the values onto M (log ratio) and A (mean average) scales. The differences in gene expression are shown on the MA plot, where genes with ≥1-fold expression values are shown in red and those with negative log2 fold values are shown in green (Figure S2).

### 2.4. Identification of Transcription Factor Genes

Transcription factors play crucial roles in regulating gene expression, and many TFs control ABGs [30]. Hence, we searched for TFs that regulate anthocyanin biosynthesis in *B. rapa*. We detected 1625 TF genes in 54 TF families from our transcriptome data (Figure 2d; Table S4 and Table S5). The proportions of TF genes in different TF families were as follows: 8.7% in the basic helix loop helix (bHLH) family, 7.6% in the ethylene response factor (ERF) family, 6.05% in the myeloblastosis (MYB) family, 5.77% in the WRKY family, and 5.72% in the MYB-related family (Figure 2d). Additionally, 218 TF genes with log2fold change expression ≥1 and 37 TFs with log2fold change expression ≤ 1 were identified (Table S4 and Table S5). A few TF genes had very high log2fold change values (5–11.3), including *MYB90 (Bra004162), MYB75 (Bra039763),* three *RRTF1s (Bra017656, Bra011529, Bra034624), CBF4 (Bra028290), MYBL2 (Bra016164), TTG2 (Bra023112), DDF1 (Bra019777)* and *ERF-13 (Bra037630)* (Table 3). These DEGs may be involved in various functions related to pigment accumulation in RPCC.

### 2.5. Functional Characteristics of DEGs and KEGG Pathway Enrichment Analysis

**Figure 3:**
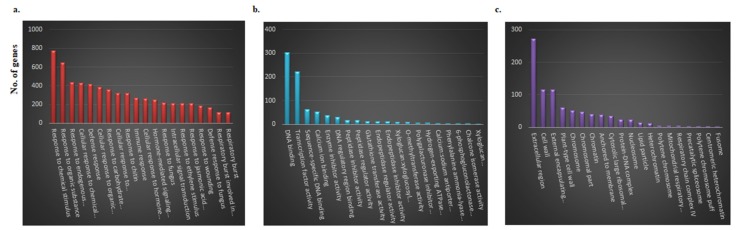
Gene ontology (GO) analysis of differentially expressed genes (DEGs) between red and green Chinese cabbage. DEGs were grouped into three categories: (**a**) Biological process; (**b**) molecular function; and (**c**) cellular component. X-axis shows gene annotation term; y-axis shows number of genes.

We performed GO analyses to annotate the DEGs. The top 20 enriched terms in each GO category of DEGs were selected based on significance (*p* < 0.05) and are summarized in Figure 3. In the BP category, the majority of DEGs were involved in “response to chemical stimulus” followed by “response to organic substance”, “response to endogenous stimulus”, and “cellular response to chemical stimulus” processes. Similarly, in the MF category, the majority of genes were involved in “DNA binding”, followed by “transcription factor activity”, “sequence specific DNA binding” and “calcium ion binding”. In the CC category, many of the DEGs were related to “extracellular region”, “cell wall”, “external encapsulating structure” and “plant type cell wall” (Figure 3). We further categorized the up-regulated and down-regulated genes. Among the up-regulated DEGs, a total of 385 GO terms were identified, comprising 328 in the BP category, 35 in the MF category, and 22 in the CC category. Among the down-regulated DEGs, a total of 210 GO terms were identified, comprising 133 terms in the BP category, 50 in the MF category, and 22 in the CC category (Table S6.) In the BP category, a few genes were involved in “biosynthetic and metabolic processes of anthocyanins, ethylene signaling, flavonoids and phenylpropanoids”, “catabolic and metabolic processes of L-phenylalanine”, “cinnamic acid biosynthesis”, “response to absence of light”, and “response to temperature stimulus”. In the MF category, some genes were involved in “phenylalanine ammonia-lyase activity” and “O-methyltransferase activity” (Table S6.). Some of the down-regulated genes were involved in “biosynthesis process”, “negative regulation of cellular metabolic process”, and “response to UV-C” (Table S6).

**Figure 4:**
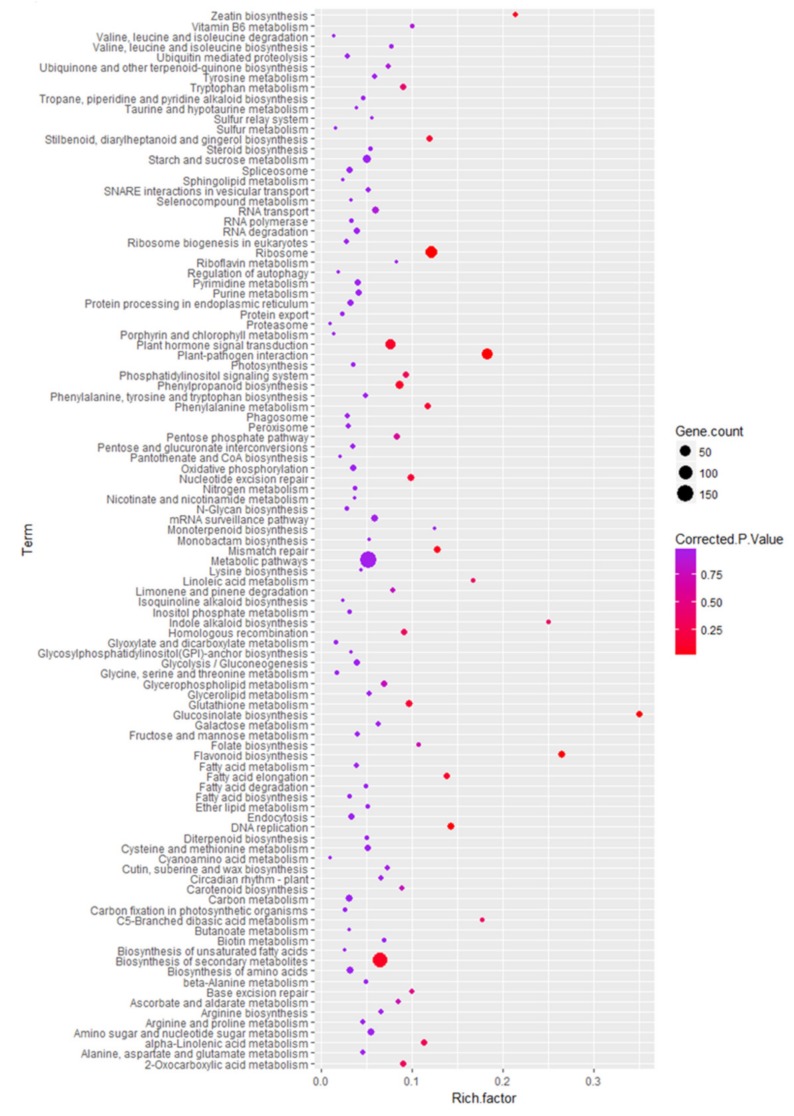
KEGG enrichment analysis of differentially expressed anthocyanin biosynthetic genes. *X*-axis shows KEGG terms and y-axis shows enrichment factor. Gene count and corrected *p*-values are shown on right.

Next, we conducted a KEGG enrichment analysis to identify pathways significantly enriched with DEGs. The pathways enriched with up-regulated DEGs were “biosynthesis of secondary metabolites”, “metabolic pathways”, “biosynthesis of flavonoids and phenylpropanoids”, “metabolism of fructose, mannose, starch, and sucrose”, and several others (Figure 4 and Table S7). The down-regulated DEGs were involved in 46 types of functions related to organ development, and other functions related to plant growth and development (Table S7).

### 2.6. Genes Related to Anthocyanin Biosynthesis Identified from Transcriptome Data

From the transcriptome data, we identified 255 ABGs (Table S8) comprising 58 phenylpropanoid biosynthetic genes (PBGs), 56 early biosynthetic genes (EBGs), 67 late biosynthetic genes (LBGs), 19 anthocyanin transporter genes (ATGs), 29 other anthocyanin biosynthesis regulatory genes (OABRGs) and 26 regulatory TF genes (Table S8). The main PBGs were those encoding cinnamyl alcohol dehydrogenase (CAD), caffeoyl CoA O-methyltransferase (CCoAMT), cinnamoyl-CoA reductase (CCR), cinnamate 4-hydroxylase (C4H), 4-coumarate: coenzyme A ligase (4CL), O-methyltransferase (OMT), and phenylalanine ammonia lyase (PAL). These genes are involved in different stages of the phenylpropanoid biosynthesis pathway and showed large differences in transcript levels (−0.7 to 3.2-fold change) between RPCC and GCC (Table S8). Similarly, EBGs encoding chalcone isomerase (CHI), chalcone synthase (CHS), flavanone-3-hydroxylase (F3H), flavonoid 3′-hydroxylase (F3′H), and flavonol synthase (FLS) showed large differences in their transcript levels between RPCC and GCC (−1.1 to 4.9-fold change) (Table S8). The LBGs showing large differences in transcript levels (−1.9 to 9.3-fold change) between RPCC and GCC encoded beta glucosidase (BGLU), dihydroflavonol 4-reductase (DFR), leucoanthocyanidin dioxygenase (LDOX), 5-*O*-glucoside-6-*O*-malonyltransferase (5MAT), UDP-glucose: flavonoid 3-o-glucosyltransferase (UF3GT), and UDP-glucosyltransferases (UGT). Genes for anthocyanin transporters and regulatory TFs included those encoding glutathione S-transferase 26/TRANSPARENT TESTA 19 (GST26/TT19), multidrug and toxic compound extrusion (MATE), basic helix-loop-helix 32 (BHLH32), ENHANCER OF GLABRA 3 (EGL3), GLABRA 3 (GL3), myeloblastosis protein 75 (MYB75), myeloblastosis protein 90 (MYB90), TRANSPARENT TESTA 8 (TT8) and TRANSPARENT TESTA GLABRA 1 (TTG1), and TRANSPARENT TESTA GLABRA (TTG2). These genes showed differences in expression between RPCC and GCC ranging from a −0.5 to 11.3-fold change. Interestingly, we identified a few OABRGs with high log2fold expression values (≥ 2) (Table S8). These results indicate that ABGs have vital roles in anthocyanin biosynthesis, transportation, and accumulation in the leaf tissues of RPCC at the seedling stage (3 weeks).

### 2.7. Expression Analysis of Anthocyanin Biosynthetic Genes by qRT-PCR

In general, gene expression studies can demonstrate the biological activity of genes in plants. We confirmed the reproducibility and accuracy of DEGs identified in our transcriptome data through qRT-PCR analyses. Although transcriptome sequencing was performed using samples from 3-week-old plants (seedlings), we checked the expression profiles of genes in samples from 6-week-old (rosette stage) and 9-week-old (heading stage) plants of GCC and RPCC. This analysis of gene expression at three developmental stages sheds light on the expression profile of ABGs and their effect on anthocyanin biosynthesis and accumulation throughout plant development. In general, the anthocyanin biosynthesis pathway can be classified into three phases; 1. The phenylpropanoid pathway; 2. Early steps of the flavonoid pathway; and 3. The anthocyanin pathway [31]. For the validation of gene expression, we selected 60 genes identified from the transcriptome data and from a previous study [26] that are involved in various stages of anthocyanin biosynthesis (Table 4; Table S9).

**Figure 5:**
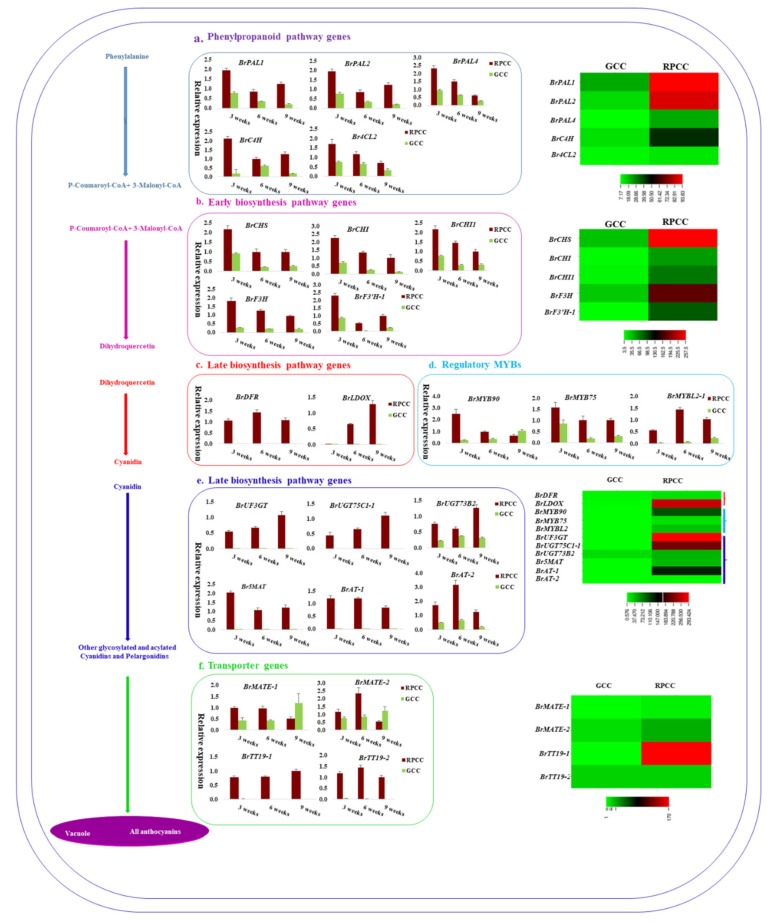
Validation of anthocyanin biosynthetic genes (ABGs) detected in transcriptome data by qRT-PCR analyses of reddish purple (RPCC) and green (GCC) leaf tissue samples. (**a**) Phenylpropanoid pathway genes; (**b**) early biosynthesis pathway genes; (**c**) and (**e**) late biosynthesis pathway genes; (**d**) regulatory MYB genes; and (**f**) transporter genes. Gene expression levels were normalized against that of *Actin*. Error bars are based on mean of three technical replicates. Schematic representation of anthocyanin biosynthetic pathway is shown in left corner. Heatmaps in middle and right corner indicate transcript abundance of ABGs.

In the phenylpropanoid pathway, three PAL genes (*BrPAL1, BrPAL2* and *BrPAL4*), *BrC4H*, and the 4CL homolog *Br4CL2* were detected in GCC and RPCC at three developmental stages. The transcriptome data from 3-week-old plants (heat map, Figure 5a) and the qRT-PCR analyses showed that the transcript levels of these genes were much higher in RPCC than in GCC. Gene expression was also compared among stages (seedling, rosette, and heading stages) (Figure 5a). Similar to PBGs, EBGs such as *BrCHS*, *BrCHI* and *BrCHI1, BrF3H,* and *BrF3′H-1* showed similar expression patterns in both the transcriptome analysis (heat map, Figure 5b) and qRT-PCR analyses (Figure 5b). Unlike PBGs and EBGs, the LBG *BrDFR* was expressed only in the RPCC at all stages, while the LBG *BrLDOX* was expressed at lower levels at the early stage. The *BrLDOX* transcript levels gradually increased from the rosette to the heading stage in RPCC (Figure 5c). The transcript levels of MYBs including *BrMYB90, BrMYB75, and BrMYBL2-1* varied among different stages. *BrMYB90* and *BrMYB75* showed maximum transcript levels in RPCC at the seedling stage, while *BrMYBL2-1* had higher transcript levels at the rosette and heading stages than at the seedling stage in RPCC (Figure 5d). 

The downstream LBGs are involved in the acylation, glycosylation, and methylation of anthocyanins and include genes encoding acyltransferase (AT), glycosyltransferase (GT) and O-methyltransferase (OMT) [32,33]. The downstream LBGs selected from the transcriptome data for qRT validation included *BrUF3GT*, *BrUGT75C1-1, BrUGT73B2, Br5MAT, BrAT-1, and BrAT-2. BrUF3GT, BrUGT75C1-1*, and *BrUGT73B2* showed increased expression while *Br5MAT* and *BrAT-1* showed decreased expression from the seedling to heading stages in RPCC. The highest transcript level of *BrAT-2* was at the rosette stage in RPCC (Figure 5e). Interestingly, all the LBGs including regulatory MYB (RM) genes showed low or no expression in GCC compared with RPCC in the transcriptome data (heat map, Figure 5e) and in the qRT-PCR analyses (Figure 5e). 

Among many anthocyanin transporter genes, four *B. rapa* transporter genes including *BrMATE-1, BrMATE-2*, and *BrTT19-1* showed differential expression between GCC and RPCC (heat map, Figure 5f). At the seedling and rosette stages, both *BrMATE-1* and *BrMATE-2* were expressed at higher levels in RPCC than in GCC. *BrTT19-1* and *BrTT19-2* transcript levels were high at all stages in RPCC, but at negligible or undetectable levels in GCC at all stages (Figure 5f). The remaining ABGs, showed diverse expression patterns in the qRT-PCR analyses (Figure S3). Most of the analyzed genes had higher transcript levels in RPCC than in GCC (Figure S3).

**Figure 6:**
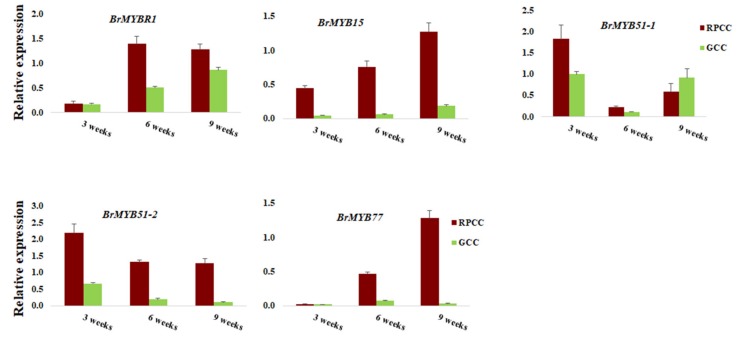
qRT-PCR validation of MYBs with high transcript abundance in transcriptome data. qRT-PCR expression values were normalized against that of *Actin*. Error bars are based on mean of three technical replicates.

We also conducted qRT-PCR analyses for some MYB TF genes showing differences in expression levels between RPCC and GCC in transcriptome data (Table S4). Among the five selected MYB genes, BrMYB15 and BrMYB51-2 showed >1-fold expression in RPCC than in GCC at all stages, and BrMYB77 transcript levels increased from the rosette to the heading stage in RPCC but not in GCC (Figure 6).

**Figure 7:**
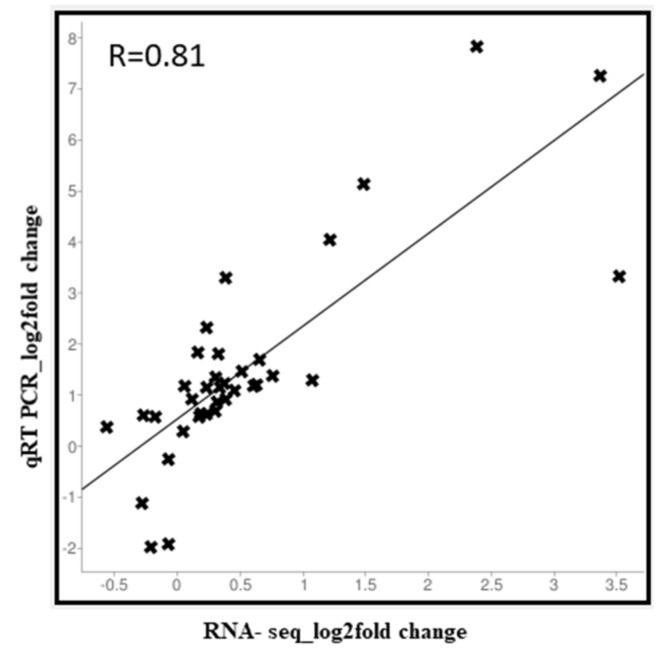
Correlation analysis between RNA-seq and qRT-PCR methods. Log2fold values of RNA-seq data (x-axis) are plotted against log2fold values of qRT-PCR (*y*-axis) data.

A correlation analysis revealed a strong correlation between the transcriptome data and qRT-PCR data (R = 0.81) (Figure 7). Overall, similar trends in gene expression were detected from transcriptome data and qRT-PCR analyses. The expression patterns of PBGs, EBGs, LBGs, TGs, and RMs implied that LBGs, TGs and RMs play crucial roles in anthocyanin biosynthesis during different developmental stages of RPCC.

### 2.8. Promoter Analysis of Anthocyanin Biosynthetic Genes

**Figure 8:**
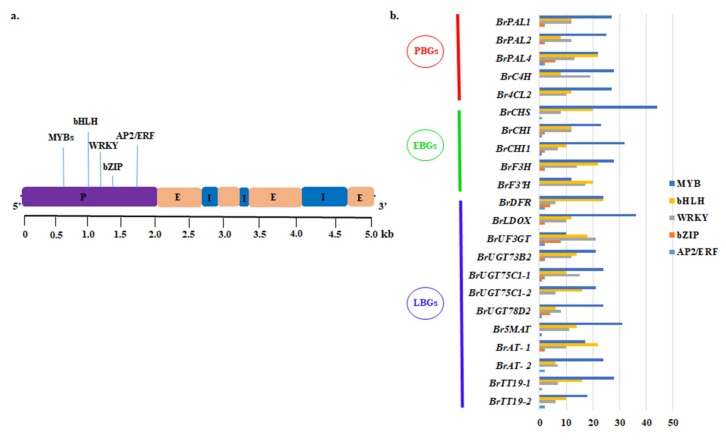
Cis-regulatory elements predicted in upstream promoter regions of anthocyanin biosynthetic genes (ABGs). (**a**) Example of plant gene organization and important cis-elements in promoter. (**b**) Number of each type of cis-element identified in ABGs. P, promoter; E, exon; I, intron.

Cis-regulatory elements (CREs) are binding sites for TFs in the promoters of target genes. To identify CREs, we analyzed the promoter regions of 22 important ABGs. The 2-kb region upstream of the transcription start site (TSS) was extracted and analyzed by the New PLACE program to find CRE motifs (Figure 8a). Among the predicted CREs, most were binding elements for MYB, bHLH, WRKY, bZIP and Ap2/ERF TFs (Figure 8b and Table S10). Those binding to MYB TFs were the most abundant, followed by those binding to bHLH, WRKY, bZIP and AP2/ERF TFs (Figure 8b). MYB CREs have been found in the promoters of genes related to secondary metabolism, flavonoid biosynthesis, anthocyanin biosynthesis, and plant defense (Table S10). The bHLH and bZIP CREs are known to be involved in the light response, tissue specific activation of phenylpropanoid biosynthetic genes, sugar repression, seed development, and the biosynthesis of phenylpropanoids, lignin, and flavonoids. AP2/ERF CREs are involved in functions related to the ethylene response, the jasmonate response, and secondary metabolism (Table S10). Therefore, the results of our study and other studies [34,35] indicate that MYB, and bHLH CREs regulate the expression of genes at all stages in the anthocyanin biosynthesis pathway.

### 2.9. Regulatory Network Analysis of Anthocyanin Biosynthesis Genes

**Figure 9:**
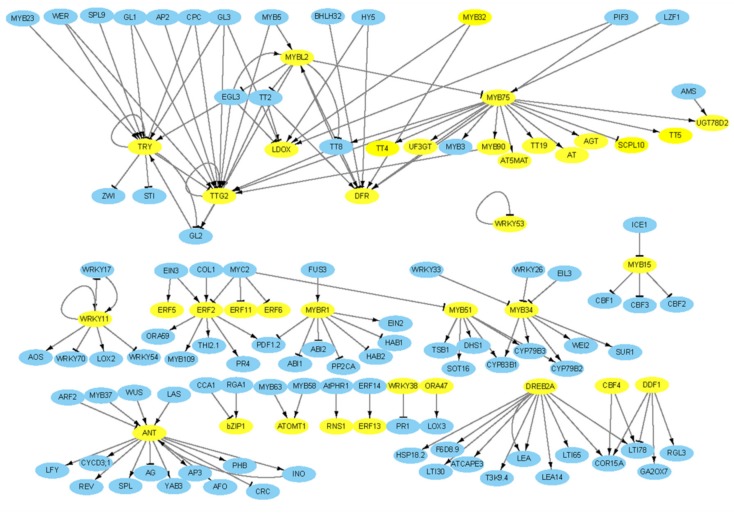
Gene regulatory network of anthocyanin biosynthetic genes and important transcription factors. DEGs detected from our transcriptome data are shown in yellow: other interactive genes involved in various functions including anthocyanin biosynthesis are shown in blue. Up tack (┴) indicates repressors and arrow (↓/↑) indicates activators.

To identify the interactions among anthocyanin biosynthetic genes and the transcription factor genes including MYB, bHLH, WRKY, bZIP, and AP2/ERF (with log2fold change > 2) (Table 3 and Table 4), a putative interactive network was constructed (Figure 9). Among them, 37 *B. rapa* genes (yellow circles) showed 147 interactions, which could be classified into two types: activation (↓/↑) and repression (┴) (Figure 9). The gene network results showed that MYB75 interacts with a gene encoding an acyl transferase family protein, as well as MYB90, 5MAT, TT5, AGT, TT4, UGT78D2, TT19, DFR, and UF3GT, and activates them to promote anthocyanin biosynthesis. Two LBGs (DFR and LDOX) are positively regulated by TFs such as PIF3, MYB32, HY5, and TT2 (Figure 9) and DFR is also positively regulated by the TT8 TF. Besides gene–gene interactions, this network analysis revealed many other interactions among TFs and structural genes involved in various functions. Among the repressors, the MYBL2 TF interacts with MYB75, DFR, TT2, TT8, GL2, and EGL3; MYB75 represses SCPL10; both EGL3 and GL3 repress LDOX; and bHLH32 represses DFR (Figure 9). These results indicate that interactions between TFs and their target genes play a vital role in the regulation of anthocyanin biosynthesis and other metabolic functions related to the growth and development of RPCC.

## 3. Discussion

The red color in Chinese cabbage has been introduced through different techniques of introgression breeding. Among the introduced varieties, Xie et al. [3] introgressed the red color phenotype by crossing a Chinese cabbage variety with red color *Brassica juncea* through the embryo rescue technique. While in our study, RPCC and red Chinese cabbage (RCC) reported by Lee et al. [4], have been developed through interspecific-crossing between Chinese cabbage and red cabbage.

### 3.1. Novel Anthocyanin Pigments Responsible for the Color of RPCC

**Figure 10:**
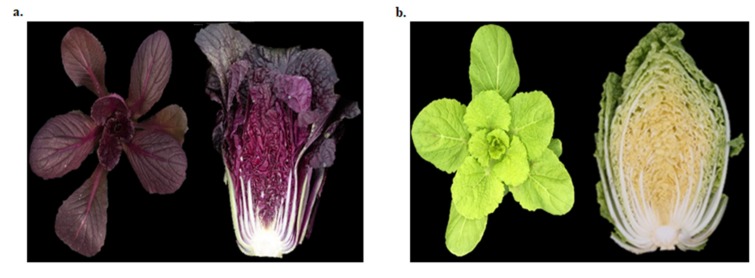
Chinese cabbage at young and mature stages. (**a**). Green (GCC) and (**b**). reddish purple (RPCC).

**Figure 11:**
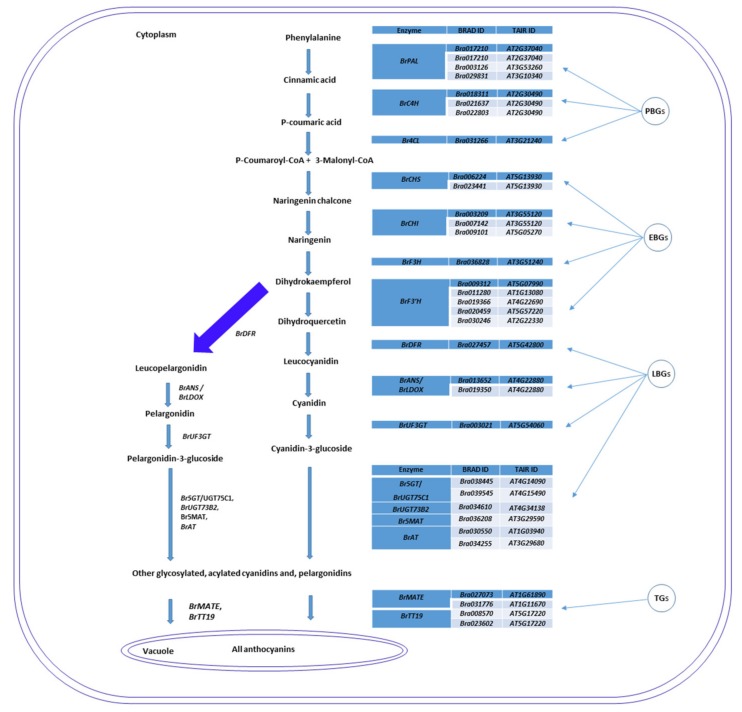
Schematic representation of anthocyanin biosynthetic pathway based on anthocyanin pigments and important anthocyanin biosynthetic genes (ABGs) identified from transcriptome data.

Red Chinese cabbage is an economically important variety that is rich in various secondary metabolites including anthocyanins [4]. This variety accumulates red pigments at an early stage of plant growth (seedling stage), so it is an ideal system to study the genes and regulatory TFs that are involved in regulating color (anthocyanin) accumulation at the early stages of plant development (Figure 10). Fruits and vegetables contain five main types of anthocyanins, with different frequencies of occurrence: cyanidin (50%), delphinidin (12%), pelargonidin (12%), peonidin (12%), malvidin (7%), and petunidin (7%) [36]. We detected 11 anthocyanin variants in the RPCC samples, approximately 85% of which were cyanidin isoforms. Thus, our results and those of other studies show that cyanidin derivatives are the most abundant type of anthocyanins in red Chinese cabbage [4]. In addition, the anthocyanin pigments accumulated in RPCC and in previous studies [3,4] are entirely different, indicating that the color accumulation and regulation are due to different sets of anthocyanin pigments. In accordance with the results of the HPLC analysis, we propose a model of the anthocyanin biosynthesis pathway that generates cyanidin 3-(feruloyl) diglucoside-5-(malonoyl) glucoside, and pelargonidin 3-(caffeoyl) diglucoside-5-(malonoyl) glucoside in RPCC (Figure 11). Most of the anthocyanin components identified in this study have been detected in radish or other *Brassica* crops but not in red Chinese cabbage [20,21,22,23,37,38,39,40,41,42,43]. Hence, 11 of the 13 pigments identified in our study were detected for the first time in RPCC (Table S1). Joo et al [28] have proved that the anthocyanin rich extract has the ability to lower the risk of vascular inflammatory diseases.

### 3.2. Anthocyanin Biosynthesis Genes Are Differentially Regulated in RPCC

Transcriptome sequencing is an advanced NGS technique that can be used to predict novel genes, gene function, and genome evolution. Comparative transcriptome sequencing between two different phenotypes, GCC and RPCC, revealed differences in the expression levels of genes involved in anthocyanin biosynthesis and the regulation of this pathway [26,44]. The transcriptome sequencing of RPCC and GCC at the seedling stage revealed 3345 DEGs, which included unique genes with unknown functions and TF genes. About 255 DEGs were involved in various functions related to phenylpropanoids, lignins, flavonols, and anthocyanins. Further qRT-PCR analyses showed that PBGs and EBGs were expressed at levels 0.5- to 1.0-fold higher at the seedling stage than at the other two stages (rosette and heading) in RPCC. Many of these genes encoded proteins involved in the early phases of anthocyanin biosynthesis [7,45,46]. Our results indicate that *BrPAL, BrPAL2, BrPAL4, BrC4H, Br4CL2, BrCHS, BrCHI, BrCHI1, BrF3H,* and *BrF3′H-1* may be involved in the early phase of anthocyanin biosynthesis (i.e., the conversion of phenylalanine to dihydroquercetin) in RPCC. As shown in Figure 5e, the important LBGs *BrDFR* and *BrLDOX*, whose encoded products catalyze the conversion of dihydroquercetin to cyanidin at the late stage of anthocyanin biosynthesis, were expressed in RPCC at all stages, but not in GCC at any stage. Previous studies have shown that DFR plays crucial roles in anthocyanin accumulation in many plant species under different abiotic stress conditions [47,48]. Similarly, in *Arabidopsis,* sucrose and jasmonic acid have been shown to induce LBGs such as *DFR, LDOX*, and *UF3GT*, leading to anthocyanin accumulation [45,49]. Interestingly, we detected high transcript levels of the LBGs *BrDFR, BrLDOX*, and *BrUF3GT* in RPCC but not in GCC, indicating that anthocyanin biosynthesis occurs in RPCC under normal growth conditions without induction by external factors such as sugars or hormones.

Some downstream LBGs are involved in p-coumaroylation (*At3AT1*: At1g03940), glucosylation (*UGT75C1*:At4g14090) and malonylation (*At5MAT*: At3g29590) [33,50,51,52]; the orthologs of these genes (*BrAT-1, BrUGT75C1*-*1*, and *Br5MAT*) were expressed only in RPCC. Accordingly, the HPLC analyses detected p-coumaroyl (*At3AT1*: At1g03940) diglucoside (*UGT75C1*: At4g14090), indicating that anthocyanins have been modified as reported previously [33]. Our qRT-PCR analyses showed that the transcript levels of *BrUGT73B2* and *BrAT-2*, whose encoded proteins catalyze p-coumaroylation and glucosylation, respectively, were much higher in RPCC than in GCC at the rosette and heading stages. TT19 encodes a transporter involved in the movement and accumulation of anthocyanins in the Brassicaceae [53,54]. In this study, two TT19 paralogs, *BrTT19-1* and *BrTT19-2,* which have a common ortholog in *A. thaliana* (*AtTT19*: AT5G17220), showed very high transcript levels in RPCC, indicating that transport of anthocyanins from the cytosol to the vacuole is an important process in RPCC.

### 3.3. Differential Expression of MYBs Regulates Reddish Purple Color Accumulation

In general, ABGs are controlled by various TFs, including the MYB, bHLH, and WD40 TFs that make up the most well-known complex in plants, the MBW complex [55,56]. A study using the transcriptome approach in red Chinese cabbage identified that the anthocyanin pathway regulating genes are TT8 (*Bra037887*) and PAP1 (*c3563g1i2*) [3]. Most TFs that are known to regulate anthocyanins are MYB TFs. A study on grapes reported that the MYBA transcription factor regulates the anthocyanin biosynthesis pathway through controlling the expression of *UFGT* [57]. Because TFs, especially MYB, are known to be important for controlling expression of ABGs, we searched our transcription data for TF sequences. As a result, we identified 25 TF genes with log2fold change > 3: 12 TF genes with log2fold change > 4, five TF genes with log2fold change > 5 and > 6, and one TF gene with log2fold change > 10 (Table 3). They included two important MYB genes: *MYB90* (log2fold change, 11.3) and *MYB75* (log2fold change, 6.8), which are homologs of *PRODUCTION OF ANTHOCYANIN PIGMENT 2 (PAP2)* and *PRODUCTION OF ANTHOCYANIN PIGMENT 1 (PAP1)*. These genes showed similar transcript levels in qRT-PCR and transcriptome analyses, and their high expression levels in RPCC suggested that they might be involved in the positive regulation of LBGs during anthocyanin biosynthesis [13,58]. We identified duplicate copies of *MYBL2, Bra016164 (BrMYBL2-1)* and *Bra007957 (BrMYBL2-2),* in *B. rapa*, which are orthologs of *A. thaliana AtMYBL2* (*AT1G71030*). The transcript levels of both *BrMYBL2-1* and *BrMYBL2-2* were very high, as revealed by transcriptome analysis (log2fold change of 5.5 and 4.06, respectively) and qRT-PCR analysis. A previous study detected a similar expression pattern of *MYBL2* in *B. rapa,* indicative of its role as a positive regulator [59]. However, other studies on the Brassicaceae have demonstrated that MYBL2 can function as a negative regulator of anthocyanin biosynthesis [60,61]. Functional characterization of *BrMYBL2-1* and *Bra007957* (*BrMYBL2-2*) will clarify the molecular mechanisms of these genes in RPCC. Our results also showed that MYB binding motifs are highly conserved not only in LBGs but in most of the analyzed ABGs.

## 4. Materials and Methods

### 4.1. Plant Material and Sample Collection

The RPCC was developed (through introgression hybridization) and registered by the Kwonnong Seed Company (Cheongju, S. Korea) as described by Lee et al. [4]. The lines used in this study, green Chinese cabbage (GCC) and RPCC, belong to *B. rapa* L. ssp. *pekinensis* and were selected on the basis of their distinct color phenotypes (Figure 1). Seeds of GCC and RPCC were germinated in a growth chamber under a 16-h light/8-h dark photoperiod at 24 °C. Leaf samples (innermost and outermost leaves) were collected from three biological replicates at three growth stages: the seedling, rosette, and heading stages (at 3-, 6-, and 9-weeks-old, respectively). The leaf samples were stored at −70 °C until further analysis.

### 4.2. Anthocyanin Extraction and HPLC Analysis

The total anthocyanin content of freeze-dried outer and inner leaf tissues of 9-week-old GCC and RPCC was determined by HPLC and LC-MS/MS, as described previously [37]. Each 100-mg lyophilized leaf sample was mixed with 2 mL water:formic acid (95:5 *v*/*v*) followed by 5 min vortexing and 20 min sonication. The sample was centrifuged at 9200× *g* for 15 min at 4 °C and the supernatant was filtered through a 0.45-µm PTFE hydrophilic syringe filter. From this filtrate, 10 μL was used for estimating anthocyanin content. The sample was injected into an Agilent 1200 series HPLC connected to a 4000 Q-Trap LC-ECI-MS/MS system. A Synergy 4µL Polar-RP 80A column (250 × 4.6 mm i.d., particle size 4 µm; Phenomenex, Torrance, CA, USA) with a Security Guard Cartridge (AQ C18, 4 × 3.0 mm KJO-4282; Phenomenex, Torrance, CA, USA) were used. Anthocyanin pigments were detected at 520 nm. The oven temperature was set to 40 °C. The composition of the mobile phase was as follows: solvent A: water:formic acid (95:5 *v*/*v*), and solvent B (acetonitrile:formic acid, 95:5 *v*/*v*). The gradient conditions were as follows: 0–8 min, 8–13 min, 13% solvent B; 13–20 min, 20–23 min, 17% solvent B; 23–30 min, 30–40 min, 20% solvent B; 40–40.1 min, 5% solvent B; and 40.1–50 min, 5% solvent B. The anthocyanin concentration in each sample was measured by comparison of the area of each peak with that of the external standard (cyanidin-3-*O*-glucoside) on the HPLC chromatogram. Mean ± SD values were calculated from the three replicates of each sample.

### 4.3. Sequence Pre-Processing and Assembly

For transcriptome analysis, total RNA was extracted from leaf tissues of 3-week-old plants using an RNeasy Mini kit (Qiagen, Valencia, CA, USA) and sequenced with the Illumina Hi-seq2000 platform by SEEDERS Inc. (Korea). The sequence data was submitted to the NCBI database and are available under the accession number PRJNA612946. The raw reads were trimmed using the Dynamic-Trim and Length-Sort programs of the Solexa QA [62] package. Based on the Dynamic-Trim (phred score ≥ 20) and Length-Sort (short read length ≥ 25bp) parameters, clean reads were obtained. The clean reads were assembled according to the protocols of Velvet (version 1.2.08) and Oases (version 0.2.08) software [63]. The optimal k-mer was selected based on the max length, average length, and N50 according to the total length of the assembled sequence.

### 4.4. Mapping and Annotation of Transcripts

To identify gene function, the transcripts were used as queries in BlastX searches against the amino acid sequences in the BRAD [64] and KEGG databases with the following parameters: filter criterion: e-value ≤ 1e−10, best hits. Mapping was performed using Bowtie2 (v2.1.0) software with the following limitation: mismatch ≤ 2 bp, computed by the penalty method) [65]. Transcript levels were normalized using the R package of DESeq [66], and this software was also used to calculate the gene expression values for each sample with data deviation.

### 4.5. Identification of Transcription Factors

To identify TFs, sequences of all *B. rapa* 4127 TFs were downloaded from the Plant Transcription Factor Database [67] (http://planttfdb.cbi.pku.edu.cn/). The total assembled transcripts were compared and analyzed using BlastX software with the parameters e-value ≤ 1e−50 and identity ≥ 50, and annotated.

### 4.6. Prediction of Differentially Expressed Genes

Differentially expressed genes (DEGs) were defined as those with at least a log2fold difference in transcript levels between the RPCC and GCC samples. Up-regulated genes were those with log2 fold-change greater than 1, and down-regulated genes were those with log2 fold-change less than −1 [22].

### 4.7. Expression Analyses

Leaf samples collected from 3-, 6-, and 9-week-old plants were collected and immediately frozen in liquid nitrogen. Total RNA was extracted using an RNeasy Mini kit (Qiagen). cDNA was synthesized using a Reverse Transcription System kit (Promega, Madison, WI, USA). The resulting cDNA was used as a template for qRT-PCR analyses, which were conducted with the CFX96 Real-Time system (Bio-Rad, Hercules, CA, USA). qRT-PCR analyses were conducted for 60 anthocyanin biosynthetic genes (ABGs) with three biological replicates and three technical replicates using the following conditions: 95 °C for 3 min; 39 cycles of 95 °C for 15 s and 58 °C for 20 s. The relative expression levels were determined by normalizing the data with the comparative Ct method 2−[ΔΔ*C*t] [68] using the *Actin* gene as a reference.

### 4.8. Gene Ontology Annotation and KEGG Enrichment Analyses

Gene ontology (GO) [69] alignments were performed using total transcripts and ‘GO DB’ with the threshold of ‘counts ≥ 1’ and ‘GO depth’ set to 3. Genes were classified into three functional categories, BP (Biological Process), CC (Cellular Component), and MF (Molecular Function). Then, gene annotation (filter criterion: e-value ≤ 1e−10, best hits) was performed through comparisons with amino acid sequences in the Kyoto Encyclopedia of Genes and Genomes (KEGG) database using BLASTX.

To predict the functional characteristics of up- and down-regulated genes, we first computed *p*-values based on Fisher’s exact test; these values were taken as indicators of significance of the gene in the respective function. Further KEGG enrichment analysis was performed using the KOBAS online tool (http://kobas.cbi.pku.edu.cn/kobas3) [70]. These analyses provided pathway annotations for the transcripts.

### 4.9. Identification of Cis-Regulatory Elements

The 2-kb region upstream from the transcription start site of selected genes was screened to identify cis-regulatory motifs using the New PLACE web tool [71].

### 4.10. Network Analysis

We carried out network analysis to construct an active gene-to-gene regulatory network of genes positively correlated with the anthocyanin biosynthetic pathway. The STRING 10.0 database (http://string-db.org/) was used to obtain an interaction network of these genes in *B. rapa* [72], according to orthologous genes in *A. thaliana*. Every link has a score from 0 to 1, where 1 is considered as the highest confidence link for reconstruction [73].

## 5. Conclusions

In conclusion, 11 of the 13 pigments detected in RPCC are reported for the first time for this variety. Analyses of the transcriptome data from two varieties at the seedling stage revealed many unique transcripts including DEGs and TF genes that are involved in a multitude of functions in growth and development. Our results show that many DEGs between the red and green varieties are involved in the biosynthesis of secondary metabolites such as phenylpropanoids, lignins, flavonoids, and anthocyanins, and in the regulation of these biosynthetic pathways. Further qRT-PCR expression analyses confirmed that ABGs and many TFs play essential roles in anthocyanin biosynthesis. The gene-to-gene interaction network illustrates the possible regulatory mechanism of MYBs with ABGs during anthocyanin biosynthesis in RPCC. Overall, our study describes the pigments in RPCC, identifies the important anthocyanin biosynthetic genes and TF genes that control the anthocyanin biosynthesis pathway, and proposes a model for the possible interaction mechanism between ABGs and TFs.

## Figures and Tables

**Table 1:** Total anthocyanin pigments identified in outer and inner leaf tissues of reddish purple and green Chinese cabbage.

No. of Pigments	Trivial Names	RPCC_IL	RPCC_OL	GCC_IL	GCC_OL
1	Cyanidin 3-diglucoside-5-glucoside	0.26 ± 0.00	0.04 ± 0.05	ND	ND
2	Cyanidin 3-diglucoside-5-(malonyl)glucoside	1.74 ± 0.23	0.29 ± 0.23	ND	ND
3	Cyanidin 3-(feruloyl)diglucoside-5-glucoside	0.19 ± 0.01	0.00 ± 0.00	ND	ND
4	Cyanidin 3-(caffeoyl)diglucoside-5-(malonyl)glucoside	1.93 ± 0.23	0.26 ± 0.19	ND	ND
5	Cyanidin 3-(p-coumaroyl)diglucoside-5-glucoside	0.34 ± 0.03	0.13 ± 0.09	ND	ND
6	Cyanidin 3-(feruloyl)diglucoside-5-glucoside	0.76 ± 0.09	0.08 ± 0.06	ND	ND
7	Pelargonidin 3-(caffeoyl)diglucoside-5-(malonoyl)glucoside	5.66 ± 0.60	0.81 ± 0.58	ND	ND
8	Cyanidin 3-(feruloyl)diglucoside-5-(malonoyl)glucoside	12.63 ± 1.37	2.19 ± 1.60	ND	ND
9	Cyanidin 3-(feruloyl)(feruloyl)diglucoside-5-glucoside	1.25 ± 0.09	0.19 ± 0.14	ND	ND
10	Cyanidin 3-*O*-(sinapoyl)(feruloyl)diglucoside-5-*O*-glucoside	0.41 ± 0.06	0.16 ± 0.12	ND	ND
11	Cyanidin 3-*O*-(p-coumaroyl)(sinapoyl)diglucoside-5-*O*-(malonyl)glucoside	1.92 ± 0.12	0.61 ± 0.45	ND	ND
12	Cyanidin 3-*O*-(sinapoyl)(feruloyl)diglucoside-5-*O*-(malonyl)glucoside	3.55 ± 0.26	1.24 ± 0.92	ND	ND
13	Cyanidin 3-*O*-(p-coumaroyl)(sinapoyl)diglucoside-5-*O*-(malonyl)glucoside	1.68 ± 0.12	0.77 ± 0.61	ND	ND
Total		32.31 ± 2.84	10.17 ± 1.69	ND	ND

RPCC-reddish purple Chinese cabbage; GCC- green Chinese cabbage; IL-inner leaf; OL- outer leaf; anthocyanin content-mg/g dry weight.

**Table 2:** Summary of RNA sequence data.

	Reddish Purple Chinese Cabbage (RPCC)	Green Chinese Cabbage (GCC)
Total Reads	48,179,516	53,127,646
Total Bases	4866,131,116	5365,892,246
Total Bases(Gb)	4.87 Gb	5.37 Gb
GC_ Count	2320,659,939	2532,951,037
N_ Zero Reads	48,028,490	52,961,958
N5_ Less Reads	48,091,492	53,032,280
N_ Rate	0.03%	0.03%
Q20_More Bases	4699,696,370	5195,093,244
Q30_ More Bases	4579,319,019	5069,583,507
Clean reads	34,924,846	38,611,432
Clean bases	3073,838,581	3400,979,929

**Table 3:** Summary of important regulatory transcriptional factor family genes identified from transcriptome data.

BRAD ID	E-Value	Identity	TAIR Description				Log2 Fold Change
			*A. thaliana* Id	TF Family	Gene Annotation	Gene Description	RPCC vs. GCC
AP2/ERF Transcription Factors							
Bra011782	0	100	AT4G37750	AP2	*ANT, CKC, CKC1, DRG*	Integrase-type DNA-binding superfamily protein	2.21
Bra017656	0	99.88	AT4G34410	ERF	*RRTF1*	Redox responsive transcription factor 1	6.90
Bra011529	0	99.75	AT4G34410	ERF	*RRTF1*	Redox responsive transcription factor 1	6.72
Bra019087	0	100	AT2G20350	ERF	−	Integrase-type DNA-binding superfamily protein	6.50
Bra034624	0	99.8	AT4G34410	ERF	*RRTF1*	Redox responsive transcription factor 1	6.16
Bra028290	0	100	AT5G51990	ERF	*CBF4, DREB1D*	C-repeat-binding factor 4	5.94
Bra019777	0	98.04	AT1G12610	ERF	*DDF1*	Integrase-type DNA-binding superfamily protein	5.06
Bra037630	0	96.93	AT2G44840	ERF	*ATERF13, EREBP, ERF13*	Ethylene-responsive element binding factor 13	5.00
Bra015882	0	94.68	AT1G74930	ERF	*ORA47*	Integrase-type DNA-binding superfamily protein	4.96
Bra027612	0	100	AT1G63030	ERF	*DDF2*	Integrase-type DNA-binding superfamily protein	4.88
Bra016763	0	100	AT1G12610	ERF	*DDF1*	Integrase-type DNA-binding superfamily protein	4.84
Bra024539	0	99.48	AT1G22810	ERF	−	Integrase-type DNA-binding superfamily protein	4.79
Bra026963	0	98.87	AT1G12610	ERF	*DDF1*	Integrase-type DNA-binding superfamily protein	4.72
Bra031069	0	100	AT1G19210	ERF	−	Integrase-type DNA-binding superfamily protein	4.64
Bra028759	0	94.66	AT5G05410	ERF	*DREB2,DREB2A*	DRE-binding protein 2A	4.58
Bra015660	0	100	AT1G77640	ERF	−	Integrase-type DNA-binding superfamily protein	4.04
Bra028291	0	100	AT5G52020	ERF	−	Integrase-type DNA-binding superfamily protein	3.98
Bra016400	0	100	AT1G21910	ERF	−	Integrase-type DNA-binding superfamily protein	3.95
Bra032901	0	100	AT1G28370	ERF	*ATERF11,ERF11*	ERF domain protein 11	3.66
Bra014925	0	100	AT3G23230	ERF	−	Integrase-type DNA-binding superfamily protein	3.64
Bra027614	0	85	AT1G12630	ERF	−	Integrase-type DNA-binding superfamily protein	3.62
Bra002377	0	100	AT5G21960	ERF	−	Integrase-type DNA-binding superfamily protein	3.62
Bra010881	0	99.46	AT1G28360	ERF	*ATERF12,ERF12*	ERF domain protein 12	3.57
Bra016136	0	100	AT1G71450	ERF	−	Integrase-type DNA-binding superfamily protein	3.36
Bra003780	0	100	AT1G74930	ERF	*ORA47*	Integrase-type DNA-binding superfamily protein	3.29
Bra036022	0	99.44	AT1G21910	ERF	−	Integrase-type DNA-binding superfamily protein	3.28
Bra016518	3.00E−162	95.32	AT1G19210	ERF	−	Integrase-type DNA-binding superfamily protein	3.23
Bra029147	0	100	AT5G52020	ERF	−	Integrase-type DNA-binding superfamily protein	3.21
Bra024953	0	100	AT5G47230	ERF	*ATERF-5, ATERF5, ERF5*	Ethylene responsive element binding factor 5	3.14
Bra040309	0	99.15	AT1G44830	ERF	−	Integrase-type DNA-binding superfamily protein	3.14
Bra032665	0	100	AT2G44840	ERF	*ATERF13, EREBP, ERF13*	Ethylene-responsive element binding factor 13	3.00
Bra030957	0	99.3	AT1G53170	ERF	*ATERF-8, ATERF8, ERF8*	Ethylene response factor 8	2.99
Bra010880	0	98.27	AT1G28370	ERF	*ATERF11, ERF11*	ERF domain protein 11	2.91
Bra011383	0	98.7	AT4G32800	ERF	−	Integrase-type DNA-binding superfamily protein	2.91
Bra015478	1.00E−10	74.84	AT2G44840	ERF	*ATERF13, EREBP, ERF13*	Ethylene-responsive element binding factor 13	2.86
Bra021048	0	97.12	AT4G17500	ERF	*ATERF-1, ERF-1*	Ethylene responsive element binding factor 1	2.63
Bra024954	0	99.54	AT5G47220	ERF	*ATERF-2, ATERF2, ERF2*	Ethylene responsive element binding factor 2	2.61
Bra008952	0	98.76	AT5G11590	ERF	*TINY2*	Integrase-type DNA-binding superfamily protein	2.53
Bra035732	0	97.33	AT5G51190	ERF	−	Integrase-type DNA-binding superfamily protein	2.51
Bra040158	0	100	AT4G17490	ERF	*ATERF6, ERF-6-6, ERF6*	Ethylene responsive element binding factor 6	2.48
Bra017493	0	98.76	AT5G47230	ERF	*ATERF-5, ATERF5, ERF5*	Ethylene responsive element binding factor 5	2.33
Bra040159	0	100	AT4G17500	ERF	*ATERF-1,ERF-1*	Ethylene responsive element binding factor 1	2.22
Bra034535	0	100	AT4G32800	ERF	−	Integrase-type DNA-binding superfamily protein	2.11
Bra027736	0	97.6	AT1G64380	ERF	−	Integrase-type DNA-binding superfamily protein	2.00
bHLH transcription factors							
Bra033690	0	100	AT5G43650	bHLH	*BHLH92*	Basic helix-loop-helix (bHLH) DNA-binding superfamily protein	4.69
Bra027501	0	100	AT5G43650	bHLH	*BHLH92*	Basic helix-loop-helix (bHLH) DNA-binding superfamily protein	2.68
Bra035639	0	97.42	AT5G56960	bHLH	−	Basic helix-loop-helix (bHLH) DNA-binding family protein	2.21
Bra036640	0	100	AT1G62975	bHLH	−	Basic helix-loop-helix (bHLH) DNA-binding superfamily protein	2.20
bZIP transcription factors							
Bra010035	0	94.65	AT5G49450	bZIP	*AtbZIP1,bZIP1*	Basic leucine-zipper 1	3.10
C2H2 transcription factors							
Bra006692	1.00E−160	88.04	AT5G59820	C2H2	*RHL41, ZAT12*	C2H2-type zinc finger family protein	4.46
Bra002528	0	98.96	AT5G59820	C2H2	*RHL41, ZAT12*	C2H2-type zinc finger family protein	3.43
Bra022436	0	97.64	AT3G19580	C2H2	*AZF2, ZF2*	Zinc-finger protein 2	2.99
Bra010922	0	99.57	AT1G27730	C2H2	*STZ, ZAT10*	Salt tolerance zinc finger	2.96
Bra001752	0	99.87	AT3G19580	C2H2	*AZF2, ZF2*	Zinc-finger protein 2	2.96
Bra032845	0	100	AT1G27730	C2H2	*STZ, ZAT10*	Salt tolerance zinc finger	2.81
Bra038219	0	98.11	AT3G19580	C2H2	*AZF2, ZF2*	Zinc-finger protein 2	2.50
C3H transcription factors							
Bra000170	1.00E−156	100	AT2G40140	C3H	*ATSZF2, CZF1, SZF2, ZFAR1*	Zinc finger (CCCH-type) family protein	2.77
Bra007205	0	98.79	AT3G55980	C3H	*ATSZF1, SZF1*	Salt-inducible zinc finger 1	2.41
Bra004982	4.00E−135	81.1	AT2G40140	C3H	*ATSZF2, CZF1, SZF2, ZFAR1*	Zinc finger (CCCH-type) family protein	2.16
Dof transcription factors							
Bra014297	0	98.01	AT1G51700	Dof	*ADOF1, DOF1*	DOF zinc finger protein 1	2.18
GRAS transcription factors							
Bra021063	0	99.75	AT4G17230	GRAS	*SCL13*	SCARECROW-like 13	2.06
Bra033813	0	91.33	AT3G46600	GRAS	−	GRAS family transcription factor	2.02
HD-ZIP transcription factors							
Bra005259	0	100	AT2G36610	HD-ZIP	*ATHB22, HB22*	Homeobox protein 22	3.85
Bra016300	0	100	AT1G26960	HD-ZIP	*AtHB23, HB23*	Homeobox protein 23	2.02
LBD transcription factors							
Bra021433	5.00E-124	83.53	AT3G02550	LBD	*LBD41*	LOB domain-containing protein 41	2.90
MADS transcription factors							
Bra017376	0	99.84	AT2G03710	MIKC_ MADS	*AGL3,SEP4*	K-box region and MADS-box transcription factor family protein	2.33
Bra024521	0	99.59	AT1G22590	M-type_ MADS	*AGL87*	AGAMOUS-like 87	2.10
Bra005166	0	100	AT2G28700	M-type_ MADS	*AGL46*	AGAMOUS-like 46	2.05
MYB transcription factors							
Bra004162	2.00E−111	84.01	AT1G66390	MYB	*ATMYB90, MYB90, PAP2*	MYB domain protein 90	11.38
Bra039763	0	92.86	AT1G56650	MYB	*ATMYB75, MYB75, PAP1, SIAA1*	Production of anthocyanin pigment 1	6.87
Bra029990	0	100	AT3G50060	MYB	*MYB77*	MYB domain protein 77	3.89
Bra012910	0	100	AT3G50060	MYB	*MYB77*	MYB domain protein 77	2.82
Bra013000	9.00E−118	100	AT5G60890	MYB	*ATMYB34, ATR1, MYB34*	MYB domain protein 34	2.51
Bra016164	0	100	AT1G71030	MYB_ related	*ATMYBL2, MYBL2*	MYB-like 2	5.55
Bra007957	0	97.7	AT1G71030	MYB_ related	*ATMYBL2, MYBL2*	MYB-like 2	4.06
Bra022637	5.00E−79	89.91	AT5G53200	MYB_ related	*TRY*	Homeodomain-like superfamily protein	2.34
NAC transcription factors							
Bra008553	0	94.59	AT4G01550	NAC	*anac069, NAC069*	NAC domain containing protein 69	4.31
Bra020188	0	99.8	AT5G22380	NAC	*anac090, NAC090*	NAC domain containing protein 90	3.14
Bra006624	0	99.58	AT5G22380	NAC	*anac090, NAC090*	NAC domain containing protein 90	2.86
Bra027238	0	100	AT3G15500	NAC	*ANAC055,ATNAC3,NAC055, NAC3*	NAC domain containing protein 3	2.62
Bra037283	0	99.52	AT2G17040	NAC	*anac036, NAC036*	NAC domain containing protein 36	2.17
Bra013034	0	98.94	AT2G17040	NAC	*anac036, NAC036*	NAC domain containing protein 36	2.08
WRKY transcription factors							
Bra023112	0	99.53	AT2G37260	WRKY	*ATWRKY44, DSL1,TTG2, WRKY44*	WRKY family transcription factor family protein	5.13
Bra014426	0	99.88	AT2G46400	WRKY	*ATWRKY46, WRKY46*	WRKY DNA-binding protein 46	3.87
Bra003588	0	99.5	AT1G80840	WRKY	*ATWRKY40, WRKY40*	WRKY DNA-binding protein 40	3.81
Bra035148	0	100	AT1G80840	WRKY	*ATWRKY40, WRKY40*	WRKY DNA-binding protein 40	3.69
Bra005210	0	100	AT2G37260	WRKY	*ATWRKY44, DSL1,TTG2, WRKY44*	WRKY family transcription factor family protein	3.54
Bra035147	0	99.52	AT1G80850	WRKY	−	DNA glycosylase superfamily protein	3.53
Bra033158	0	98.49	AT4G11070	WRKY	*AtWRKY41, WRKY41*	WRKY family transcription factor	2.95
Bra020196	0	90.34	AT5G22570	WRKY	*ATWRKY38, WRKY38*	WRKY DNA-binding protein 38	2.87
Bra010032	0	99.58	AT5G49520	WRKY	*ATWRKY48, WRKY48*	WRKY DNA-binding protein 48	2.86
Bra019265	0	100	AT4G23810	WRKY	*ATWRKY53, WRKY53*	WRKY family transcription factor	2.65
Bra013731	0	99.57	AT4G23800	WRKY	−	HMG (high mobility group) box protein	2.41
Bra023998	0	100	AT4G31550	WRKY	*WRKY11*	WRKY DNA-binding protein 11	2.27
Bra031900	0	98.34	AT5G64810	WRKY	*WRKY51*	WRKY DNA-binding protein 51	2.13

**Table 4:** Details of 60 genes selected for validation by qRT_PCR analysis.

Given I.D	BRAD Id			Gene Position V 1.5				*A. thaliana* Id	Gene Annotation
	*B. rapa Id*	Identity	E-Value	Chromosome	Start	End	Strand		
Phenylpropanoid pathway genes									
***BrPAL1***	Bra017210	98.94	0	A04	16132008	16134574	-	AT2G37040	PAL1
*BrPAL2*	Bra003126	98.68	0	A07	14754085	14756786	-	AT3G53260	PAL2
*BrPAL4*	Bra029831	100	0	A05	22819640	22826303	-	AT3G10340	PAL4
*BrC4H*	Bra021637	97.23	0	A04	13688684	13690602	-	AT2G30490	C4H
*Br4CL2*	Bra031266	100	0	A05	17255035	17257878	+	AT3G21240	4CL2,AT4CL2
Early anthocyanin biosynthesis genes									
*BrCHS*	Bra006224	99.5	0	A03	2596137	2597594	+	AT5G13930	CHS,TT4
*BrCHI*	Bra007142	99.47	0	A09	29055564	29057157	-	AT3G55120	CHI,TT5
*BrCHI1*	Bra009101	100	0	A10	15229294	15230280	-	AT5G05270	CHI1
*BrF3H*	Bra036828	99.91	0	A09	27095567	27097080	+	AT3G51240	F3H,TT6
*BrF3′H-1*	Bra009312	100	0	A10	14356094	14358845	-	AT5G07990	F3*′*H,TT7
*BrF3′H-2*	Bra020459	90.02	0	A02	5846392	5848174	-	AT5G57220	F3*′*H,TT7
*BrF3′H-3*	Bra019366	99.68	0	A03	24701345	24702922	+	AT4G22690	F3*′*H,TT7
*BrF3′H-4*	Bra030246	100	0	A04	10022729	10024935	+	AT2G22330	F3*′*H,TT7
*BrF3′H-5*	Bra011280	94.06	0	A01	2958371	2960458	-	AT1G13080	F3*′*H,TT7
Late anthocyanin biosynthesis genes									
*BrDFR*	Bra027457	100	0	A09	10926334	10927890	-	AT5G42800	DFR,M318,TT3
*BrLDOX*	Bra013652	98.48	0	A01	6885692	6887113	-	AT4G22880	LDOX
*BrUF3GT*	Bra003021	99.81	0	A10	6063162	6064651	-	AT5G54060	UF3GT
*BrUGT75C1-1*	Bra038445	100	0	A08	8755970	8757334	-	AT4G14090	UGT75C1
*BrUGT75C1-2*	Bra039545	100	0	A01	11553894	11555345	+	AT4G15490	UGT75C1
*BrUGT73B2*	Bra034610	100	0	A08	11760868	11762581	+	AT4G34138	UGT73B2
*BrUGT78D2*	Bra023594	-	-	A02	3087246	3088800	-	AT5G17050	UGT78D2
*Br5MAT*	Bra036208	98.82	0	A09	1925788	1927140	-	AT3G29590	5MAT
*BrAT-1*	Bra030550	98.52	0	A08	20600205	20602987	+	AT1G03940	Acyl-transferase family protein-1
*BrAT-2*	Bra034255	100	0	A04	11806054	11806788	-	AT3G29680	Acyl-transferase family protein
Anthocyanin transporter genes									
*BrMATE2-1*	Bra031776	88.51	0	A09	36544120	36546929	+	AT1G11670	TT12
*BrMATE-2*	Bra027073	99.8	0	A09	8642073	8645111	+	AT1G61890	TT12
*BrTT19-1*	Bra008570	100	0	A10	11677671	11678470	-	AT5G17220	GST26,TT19
*BrTT19-2*	Bra023602	99.69	0	A02	3117740	3118547	+	AT5G17220	TT19
Other anthocyanin biosynthesis genes									
*BrOMT1-2*	Bra011292	99.13	0	A01	2896202	2897686	-	AT1G77520	OMT1
*BrCCR2*	Bra008438	100	0	A02	14649009	14650738	-	AT1G80820	CCR2
*BrRNS1-1*	Bra026570	99.86	0	A02	20391492	20392500	+	AT2G02990	RNS1
*BrCCoAMT*	Bra033968	100	0	A02	9548647	9549923	+	AT1G67980	CCoAMT
*BrFLS3*	Bra029212	99.68	0	A02	25974752	25976735	-	AT5G63590	ATFLS3,FLS3
*BrLAC17*	Bra006683	90.74	0	A03	4611941	4613968	-	AT5G60020	LAC17
*BrOxygenase protein*	Bra012691	100	0	A03	22738914	22741827	-	AT4G16770	oxygenase superfamily protein
*BrSCPL10-2*	Bra025601	98.52	0	A04	7905792	7908695	-	AT2G22980	SCPL10
*BrBGLU10*	Bra037647	100	0	A04	18448229	18455545	+	AT3G60120	BGLU10
*BrFLS1*	Bra022378	100	0	A05	18803675	18805312	+	AT3G19010	FLS1
*BrIRX12*	Bra005140	99.73	0	A05	3679277	3682262	-	AT2G38080	IRX12
*BrBGLU46-1*	Bra018969	99.24	0	A06	976174	979392	-	AT1G52400	BGLU46
*BrOST2*	Bra024452	96.24	9.00E−83	A06	16477147	16483736	-	AT2G18960	AHA1,HA1,OST2,PMA
*BrSCPL10-1*	Bra012153	88.53	0	A07	11924527	11938309	+	AT2G23000	SCPL10
*BrOMT1-1*	Bra012269	98.11	0	A07	11175574	11176865	+	AT1G21100	OMT1
*BrOMT*	Bra003707	100	3.00E−43	A07	17900548	17902240	+	AT1G76790	O-methyltransferase family protein
*BrCAD1-2*	Bra010819	99.59	0	A08	15846126	15848906	+	AT1G29690	CAD1
*BrCAD1-3*	Bra010879	99.78	0	A08	16130363	16132914	-	AT1G28380	CAD1
*BrCCoAOMT1*	Bra034600	100	0	A08	11803933	11805048	-	AT4G34050	CCoAOMT1
*BrCAD1-1*	Bra026804	100	0	A09	35535138	35538610	-	AT1G14780	CAD1
*BrRNS1-2*	Bra026846	100	0	A09	35723513	35724548	+	AT1G14220	RNS1
*BrCCR5*	Bra008743	100	0	A10	12472468	12475087	+	AT5G14700	CCR5
*BrBGLU46-2*	Bra002978	98.56	0	A10	6383388	6385871	-	AT5G54570	BGLU46
*BrOMT1-3*	Bra003009	100	0	A10	6154442	6160001	+	AT5G54160	ATOMT1,OMT1
Regulatory transcription factors									
*BrMYBR1*	Bra012149	100	0	A07	11952841	11953731	-	AT5G67300	MYBR1
*BrMYBL2-1*	Bra016164	100	0	A07	22386380	22387236	-	AT1G71030	MYB-like 2
*BrMYB15*	Bra001907	99.77	0	A03	19315200	19316380	+	AT3G23250	MYB15
*BrMYB51*	Bra025666	97.36	0	A06	6841688	6842966	+	AT1G18570	MYB51
*BrMYB51*	Bra016553	100	0	A08	18248352	18249890	-	AT1G18570	MYB51
*BrMYB75*	Bra039763	92.86	0	A02	8839008	8840737	+	AT1G56650	PAP1,MYB75
*BrMYB77*	Bra012910	100	0	A03	21598456	21599337	-	AT3G50060	MYB77
*BrMYB90*	Bra004162	84.01	2.00E−111	A07	20426416	20431671	+	AT1G66390	MYB90

## Supplementary Materials

Supplementary materials can be found at https://www.mdpi.com/1422-0067/21/8/2901/s1. References [74,75,76,77,78,79,80,81,82,83,84,85] are cited in Supplementary Materials. Figure S1. Statistics of non-redundant transcripts predicted in transcriptome data. (a) Length distribution of transcripts. (b) Transcripts annotated in public databases. Figure S2 MA plot of DEGs between green (G) vs. reddish purple (RP) Chinese cabbage identified from transcriptome data. X-axis shows log2fold change and y-axis shows differentially expressed genes. Red and green dots indicate up-regulated and down-regulated genes, respectively. Figure S3. Additional ABGs showing differential expression in transcriptome data validated by qRT-PCR. Expression values were normalized against that of *Actin*. Error bars are based on mean of three technical replicates. Table S1. Total anthocyanin pigments identified in reddish purple Chinese cabbage leaf samples. Table S2. Total up-regulated DEG transcripts between RPCC and GCC with annotations from BRAD and TAIR databases. Table S3. Total down-regulated DEG transcripts between RPCC and GCC with annotations from BRAD and TAIR databases. Table S4. Transcription factor genes up-regulated between RPCC and GCC samples, as identified from transcriptome data. Table S5. Transcription factor genes down-regulated between RPCC and GCC, as identified from transcriptome data. Table S6. Gene ontology (GO) annotation of DEG transcripts predicted from transcriptome data. GO annotations are shown for up-regulated genes (left) and down-regulated genes (right). Table S7. KEGG pathway annotation of DEG transcripts predicted in transcriptome data. KEGG annotations are shown for up-regulated genes (left) and down-regulated genes (right). Table S8. Genes identified from transcriptome data that are involved in phenylpropanoid and anthocyanin pathways with expression values and GO and KEGG annotations. Table S9. List of primer sequences used for qRT-PCR of anthocyanin biosynthetic genes. Table S10. *Cis*-regulatory elements (CREs) identified in upstream promoter regions of anthocyanin biosynthetic genes.

## Abbreviations

ABGs	Anthocyanin biosynthetic genes
ABP	Anthocyanin biosynthetic pathway
AP2/ERF	Apetala 2/ethylene response factor
AT	Acyltransferases
bHLH	Basic helix-loop-helix
DEGs	Differentially expressed genes
DFR	Dihydroflavonol-4-reductase
EBGs	Early biosynthetic genes
GCC	Green Chinese cabbage
HPLC	High performance liquid chromatography
LBGs	Late biosynthetic genes
LDOX	Leucoanthocyanidin dioxygenases
MATE	Multidrug and toxic compound extrusion
MYB	Myeloblastosis
5MAT	5-O-glucoside-6-O-malonyltransferase
PBGs	Phenylpropanoid biosynthetic genes
*qRT-PCR*	*Quantitative* real-time polymerase chain reaction
RPCC	Reddish purple Chinese cabbage
RPKM	Reads per kilo base per million mapped reads
TT19	Transparent Testa 19
UF3GT	UDP- glucose: flavonoid 3-*O*-glucosyltransferase
UGT75c1	UDP-glucosyltransferase 75c1
