# Supplementary figures and images for: Red Chinese Cabbage Transcriptome Analysis Reveals Structural Genes and Multiple Transcription Factors Regulating Reddish Purple Color
Source: Int J Mol Sci. 2020 Apr 21;21(8):2901. doi: 10.3390/ijms21082901 (PMC7215907; doi:10.3390/ijms21082901)

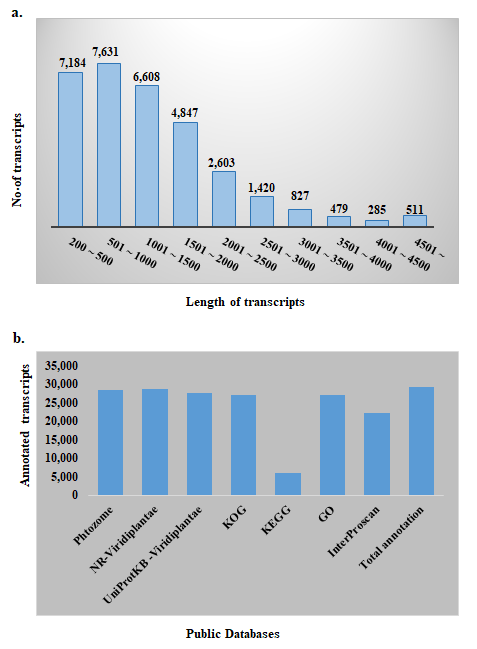

Supplement: Supplementary file 1 [file ijms-21-02901-s001.zip › Fig. S1.tif]

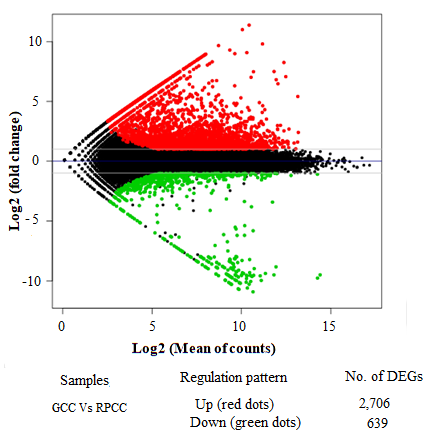

Supplement: Supplementary file 1 [file ijms-21-02901-s001.zip › Fig. S2.tif]

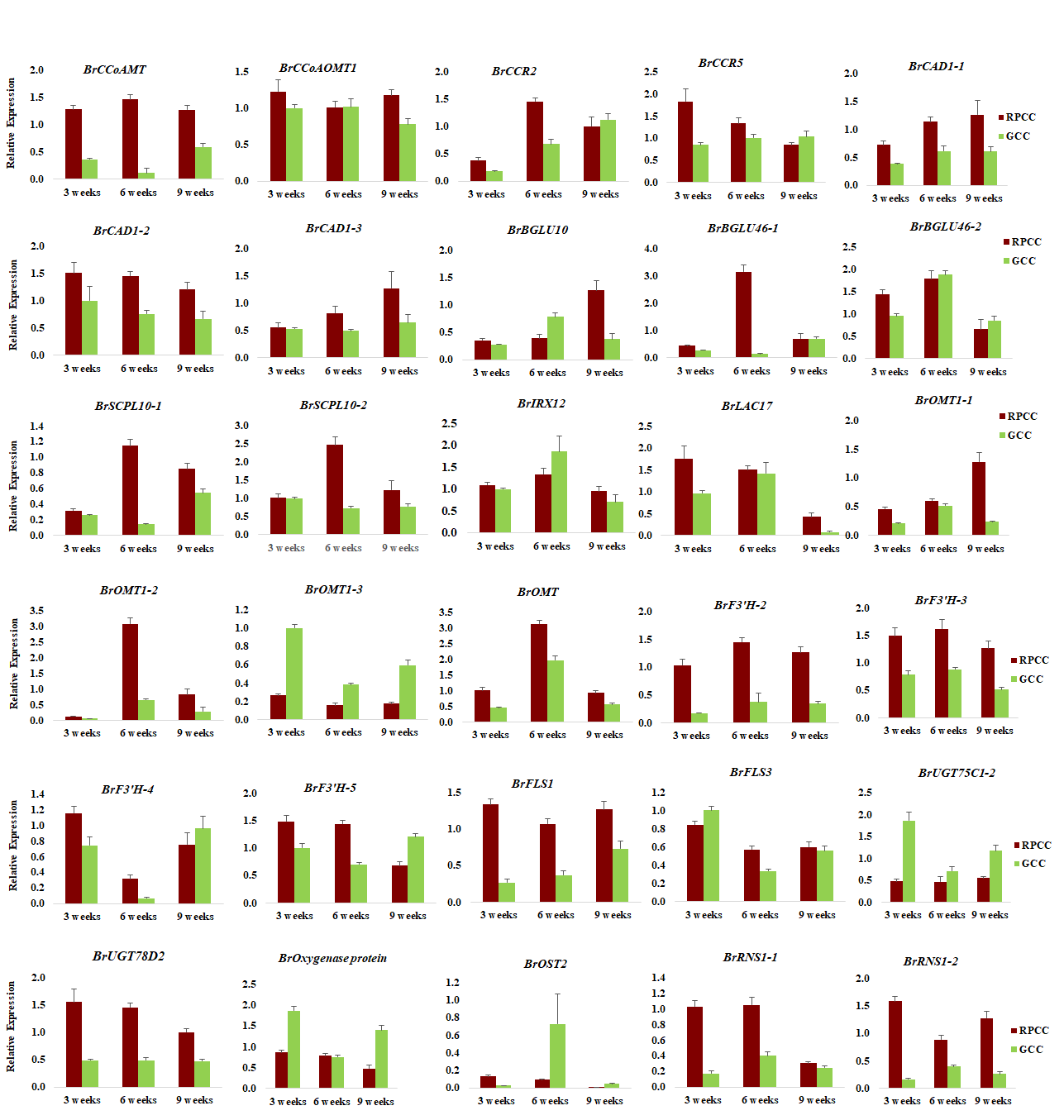

Supplement: Supplementary file 1 [file ijms-21-02901-s001.zip › Fig. S3.tif]
